# Retrospective Validation and Clinical Implementation of Automated Contouring of Organs at Risk in the Head and Neck: A Step Toward Automated Radiation Treatment Planning for Low- and Middle-Income Countries

**DOI:** 10.1200/JGO.18.00055

**Published:** 2018-08-15

**Authors:** Rachel E. McCarroll, Beth M. Beadle, Peter A. Balter, Hester Burger, Carlos E. Cardenas, Sameera Dalvie, David S. Followill, Kelly D. Kisling, Michael Mejia, Komeela Naidoo, Chris L. Nelson, Christine B. Peterson, Karin Vorster, Julie Wetter, Lifei Zhang, Laurence E. Court, Jinzhong Yang

**Affiliations:** **Rachel E. McCarroll**, **Peter A. Balter**, **Carlos E. Cardenas**, **David S. Followill**, **Kelly D. Kisling**, **Christopher L. Nelson**, **Christine B. Peterson**, **Lifei Zhang**, **Laurence E. Court**, and **Jinzhong Yang**, The University of Texas MD Anderson Cancer Center, Houston, TX; **Beth M. Beadle**, Stanford University, Stanford, CA; **Hester Burger**, **Sameera Dalvie**, and **Julie Wetter**, Groote Schuur Hospital and University of Cape Town; **Komeela Naidoo**, Stellenbosch University and Tygerberg Hospital, Cape Town; **Karin Vorster**, University of the Free State, Bloemfontein, South Africa; and **Michael Mejia**, University of Santo Tomas Hospital, Benavides Cancer Institute, Manila, Philippines.

## Abstract

**Purpose:**

We assessed automated contouring of normal structures for patients with head-and-neck cancer (HNC) using a multiatlas deformable-image-registration algorithm to better provide a fully automated radiation treatment planning solution for low- and middle-income countries, provide quantitative analysis, and determine acceptability worldwide.

**Methods:**

Autocontours of eight normal structures (brain, brainstem, cochleae, eyes, lungs, mandible, parotid glands, and spinal cord) from 128 patients with HNC were retrospectively scored by a dedicated HNC radiation oncologist. Contours from a 10-patient subset were evaluated by five additional radiation oncologists from international partner institutions, and interphysician variability was assessed. Quantitative agreement of autocontours with independently physician-drawn structures was assessed using the Dice similarity coefficient and mean surface and Hausdorff distances. Automated contouring was then implemented clinically and has been used for 166 patients, and contours were quantitatively compared with the physician-edited autocontours using the same metrics.

**Results:**

Retrospectively, 87% of normal structure contours were rated as acceptable for use in dose-volume-histogram–based planning without edit. Upon clinical implementation, 50% of contours were not edited for use in treatment planning. The mean (± standard deviation) Dice similarity coefficient of autocontours compared with physician-edited autocontours for parotid glands (0.92 ± 0.10), brainstem (0.95 ± 0.09), and spinal cord (0.92 ± 0.12) indicate that only minor edits were performed. The average mean surface and Hausdorff distances for all structures were less than 0.15 mm and 1.8 mm, respectively.

**Conclusion:**

Automated contouring of normal structures generates reliable contours that require only minimal editing, as judged by retrospective ratings from multiple international centers and clinical integration. Autocontours are acceptable for treatment planning with no or, at most, minor edits, suggesting that automated contouring is feasible for clinical use and in the ongoing development of automated radiation treatment planning algorithms.

## INTRODUCTION

When considering the need for radiation therapy (RT) in low-resource settings around the world, many challenges need to be addressed. The availability of RT resources remains the primary challenge for many regions^1,2^; however, some find that the lack of trained staff and other workflow bottlenecks, including the contouring of normal structures, are primary issues.^3^ Improving availability of RT worldwide depends not only on the deployment of equipment, but more importantly, on training staff and developing more efficient, streamlined, and practical workflows.

To address the availability of RT and simultaneous integration of high-quality techniques, there is considerable interest in the commercial development of fully automated state-of-the-art technology at an affordable cost, including intensity-modulated RT and volumetric modulated arc therapy, which offer many dosimetric advantages compared with 3D conformal and older 2D planning techniques.^4,5^ These techniques, especially critical for the treatment of head-and-neck cancer (HNC), have been shown to improve outcomes compared with 2D or 3D techniques.^6,7^ However, these techniques require significant training for optimal target and normal tissue identification, treatment planning parameters, and treatment delivery. Contouring is known to be time consuming, has considerable interphysician variability,^8-12^ and includes the component of planning, which introduces the most error.^13,14^ For physicians, the delineation of up to three prescription volumes for as many as 25 normal structures^15^ may prove daunting or simply be impossible. Opportunities to automate these steps are crucial to allowing global access to high-quality RT for HNC.

Studies into the use of automated contouring, followed by manual editing and also as a standalone contouring method, have found that for HNC treatment plans, automatic segmentation of normal structures can significantly reduce interobserver variability and contouring time.^16-18^ Authors have reported on the limited implementation of automated contouring for small structure sets (eg, brachial plexus^19^ or heart chambers^20^) and for other anatomic sites (eg, the prostate^21^). However, these uses of automated contouring require a deviation in workflow for select structures in the treatment plan. Herein, we report on the clinical implementation of a large set of automatically contoured normal structures in the head and neck.

Our objective was to retrospectively validate and assess the clinical implementation of automated contouring of eight normal structures in the head and neck as a crucial step in the development of a fully automated RT planning system for low- and middle-income countries. This included retrospective validation and the clinical implementation of normal structure autocontours generated by using an in-house autocontouring solution. To our knowledge, this is the first comprehensive assessment of automated contouring for head-and-neck structures and of the role automated contouring may play in the workflow of a typical radiation oncology clinic. Collaboration with global radiation oncologists brings to the forefront the role automated contouring will have in the practical implementation of advanced RT technologies in resource-constrained settings.

## METHODS

### Contouring Algorithm

The previously developed^22,23^ automated contouring algorithm, termed multiatlas contouring systems,^19,20^ consists of three distinct steps. First, rigid registration is performed between the test patient’s simulation computed tomography (CT) scan and the CT scans of each atlas patient using 2D sagittal and coronal projections. Second, the test patient is deformably registered to each test patient using dual-force Demons deformable registration.^24^ By using the resultant deformation vector fields, the contours from each atlas patient are mapped to the test patient,^25^ resulting in a number of individual contours equal to the number of atlas patients. Finally, the STAPLE algorithm with a built-in tissue appearance model^26^ is used to combine the individual segmentations, which generates a fusion contour approximating a true segmentation.

Central to the algorithm is an atlas of patients who are representative of the patients for which the algorithm will be used. In this work, 12 patients recently treated for HNC were selected. The atlas of normal structures included brain, brainstem, cochleae, eyes, lungs, mandible, parotid glands, and spinal cord. These structures were chosen after a small retrospective study that included other normal structures such as the esophagus, lens, optic chiasm, optic nerves, and submandibular glands. The results of that study indicated that these eight normal structures were best suited for autocontouring on the basis of comparison with independently drawn contours. The contours used for atlas building were either extracted from patient treatment plans (reviewed before treatment by a head-and-neck quality assurance peer review clinic^27^) or created by using thresholding tools. All contours were reviewed by a medical dosimetrist with 13 years of experience and a head-and-neck radiation oncologist with 8 years of experience.

### Retrospective Validation of the Automated Contouring Algorithm

#### Physician ratings.

Under an approved institutional review board protocol, the latest 128 patients stored in the database at The University of Texas MD Anderson Cancer Center and with physician-approved contours and treatment plans, were used as the study cohort. Autocontours of the eight normal structures were created and rated by an experienced head-and-neck radiation oncologist as needing no edits, needing minor edits, or needing major edits for use in dose-volume-histogram (DVH)–based planning.

To assess possible rater bias, contours from a subset of 10 patients were reviewed by five additional radiation oncologists currently treating patients with 3D conformal or volumetric modulated arc therapy techniques from four international institutions. Physician agreement was assessed by grouping each pair of ratings (one rating from the primary physician and one from an outside physician) into one of three categories. Category I agreement occurs when the physicians agreed on the degree of editing needed, and Category II agreement indicates that both physicians agreed that no more than minor edits were needed or that major edits were required. Finally, category III agreement indicates that the physicians disagreed on the acceptability of the contour, with one physician indicating that major edits were required and the other indicating that no or minor edits were required.

#### Quantitative assessment.

Autocontours were compared with independently physician-drawn contours from the patient’s original clinical plan. The Dice similarity coefficient (DSC), mean surface distance (MSD), and Hausdorff distance (HD) were measured to assess contour accuracy. DSC measures the volume overlap of the contours as a ratio to their total volume, with a minimum value of 0 and a maximum value of 1, which indicates perfect agreement. The symmetric 3D MSD between the two contours has a minimum value of 0, indicating perfect agreement and no maximum value. HD, the maximum Euclidean distance from each point in the physician contour to the nearest point in the autocontour has a minimum value of 0, indicating perfect agreement and no maximum value.

For two of the eight normal structures (lungs and spinal cord), we performed an additional quantitative analysis considering only CT slices contoured by the automated contouring algorithm. This analysis better represents the contouring accuracy of the algorithm (compared with whole-structure quantitative analysis) because it eliminates errors that arise owing to differences in CT scan extent between the test and atlas patients and among the atlas patients.

### Clinical Implementation

#### Workflow and assessment.

After retrospective validation, we began a limited introduction into our head-and-neck clinic at The University of Texas MD Anderson Cancer Center under an approved institutional review board protocol. During 10 months of clinical implementation, autocontours were generated for 166 patients. Typically, the clinical workflow of automated contouring involves initializing the algorithm by a dosimetrist who uses a script in the treatment planning system, importing the structures into the treatment plan, and reviewing which includes any needed editing of the contours by the radiation oncologist. All final contours were reviewed and edited by the attending physician in the same way that initial resident contours would be reviewed; the final contours reflected approval by the physician, with or without editing as deemed appropriate.

#### Quantitative assessment of contour edit.

To assess clinical autocontour edits, the original autocontours were compared with the contours edited for treatment planning by the physician using the DSC, MSD, and HD. In addition to analyzing the eight clinically implemented normal structures, we have also quantitatively compared edits of modified lung and spinal cord autocontours as described in the Quantitative assessment section. This provides a better estimate of the edits independent of scan and atlas extent. Generally, this is reasonable because the autocontoured structure extends inferiorly enough that it is contoured fully within the dose calculation region.

## RESULTS

### Automated Contouring Algorithm

Of the 12 patients in the contouring atlas, nine were male, 11 had primary oropharynx disease, and one patient had unknown primary cancer. The mean age was 72 years. Ten had clinical stage IVa, one had stage IVb, and one had stage III disease according to the American Joint Committee on Cancer 7th edition. All were treated with curative intent. The patient population for clinical validation primarily featured patients with oropharynx cancer (31%), oral cavity cancer (13%), and skin cancer (10%). Other primary cancer sites included hypopharynx, larynx, nasopharynx, salivary gland, and thyroid along with leukemia, lymphoma, myeloma, sarcoma, and sinonasal cancer. Ten percent of the 166 patients were younger than age 40 years, 55% were between age 40 and 65 years, and 35% were older than age 65 years. Seventy-two percent of the patients were male.

### Retrospective Validation of the Automated Contouring Algorithm

Creation of the normal structures took a mean ± standard deviation (SD) of 13.2 ± 4.1 minutes when run on a Windows 2008-based personal computer with a 6-core Xeon E5680 3.33-GHz central processing unit and 24 GB of memory. Multithread computing was enabled, which allowed two registration tasks to be run simultaneously.

#### Physician ratings.

**Fig 1:**
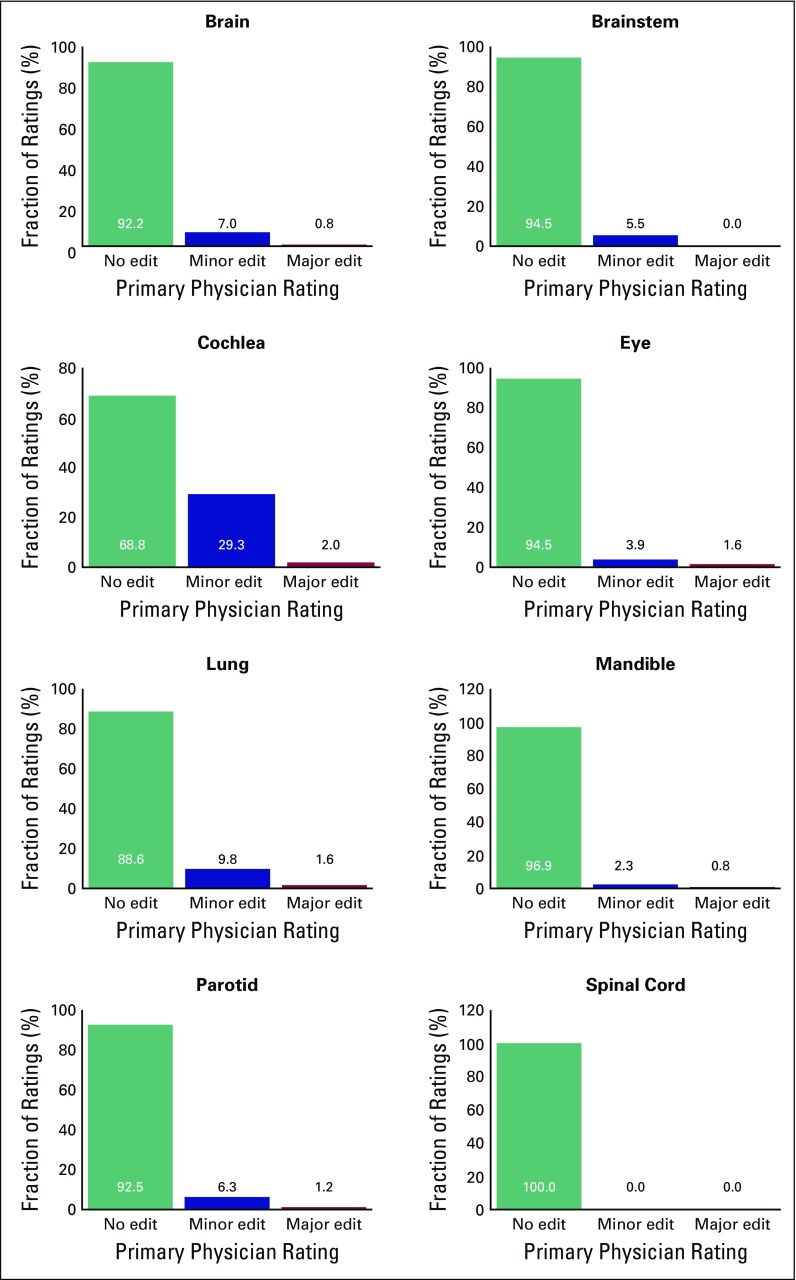
Distribution of the primary physician ratings of the eight automatically contoured normal structures (128 patients). One patient had a surgically removed parotid; for five patients, the lungs were not visible in the patient computed tomography scan, thus no rating was recorded for these structures. The mean physician ratings are displayed in the graphs.

Of the eight normal structures, on average, six were retrospectively rated as clinically acceptable for use in DVH-based planning without edits. The remaining two structures had ratings that indicated the need for minor edits for use in DVH-based planning. For all normal structures, 87% received ratings that indicated no need for edits for use in treatment planning. Furthermore, 97% of normal structures received a rating that indicated a need for only minor editing if the structure was near a target volume. Details of the score distribution by structure are provided in Figure 1.

#### Interobserver variability.

For a subset of 10 randomly selected patients, five additional radiation oncologists from four international institutions evaluated the normal-structure autocontours. The radiation oncologists (according to a self-reported questionnaire) had an average of 8.25 years of experience (range, 3.0 to 12.5 years) and contoured and/or reviewed an average of seven patients per week (range, two to 15 patients), spending an average of 95 minutes per patient on contouring (range, 45 to 180 minutes).

For all structures, 45% (245 of 547) of the ratings by the outside physicians matched those of the primary physician (Category I agreements). Considering Category II agreements, the physicians assigned an additional 48% (262 of 547) of the contours to the same group, either as needing no or minor edits for use (48%) or as needing major edits for use (0%). Finally, only 7% of contours received Category III agreement, indicating disagreement between the two ratings. The percentage of each contour classified into each of the three categories is provided in Table 1.

**Table 1:**
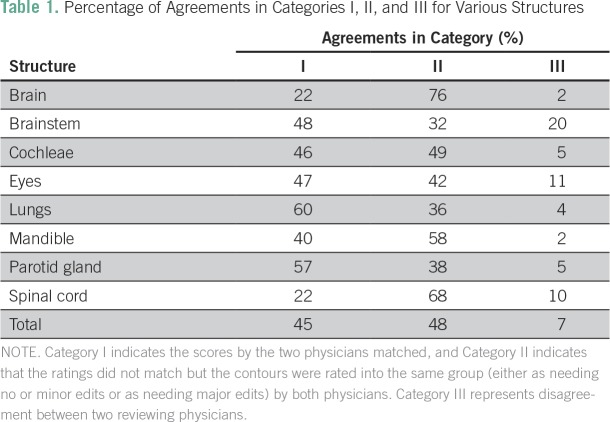
Percentage of Agreements in Categories I, II, and III for Various Structures

#### Retrospective assessment of quantitative contour accuracy.

Quantitatively, normal structure autocontours were compared with structures drawn independently by a radiation oncologist using the DSC, MSD, and HD. The DSC was greater than 0.75 for all structures except the cochleae, lungs, and spinal cord, and the MSD was less than 3 mm for all structures except the lungs and spinal cord. In these two structures, the largest difference between the autocontour and physician-drawn contour occurred at the inferior extent and was often contributable to a difference in contouring below the target volume and out of dose calculation range; considering the modified structures, the DSC and MSD improved. Table 2 lists the mean ± SD for DSC, MSD, and HD for all structures, including the two modified structures. No correlation between physician score and quantitative agreement metrics was found.

**Table 2:**
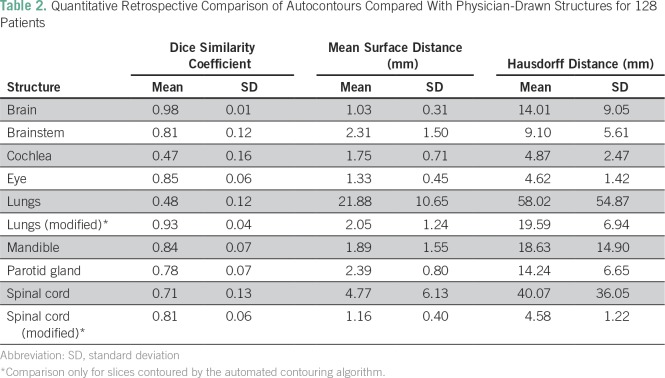
Quantitative Retrospective Comparison of Autocontours Compared With Physician-Drawn Structures for 128 Patients

### Clinical Implementation

**Fig 2:**
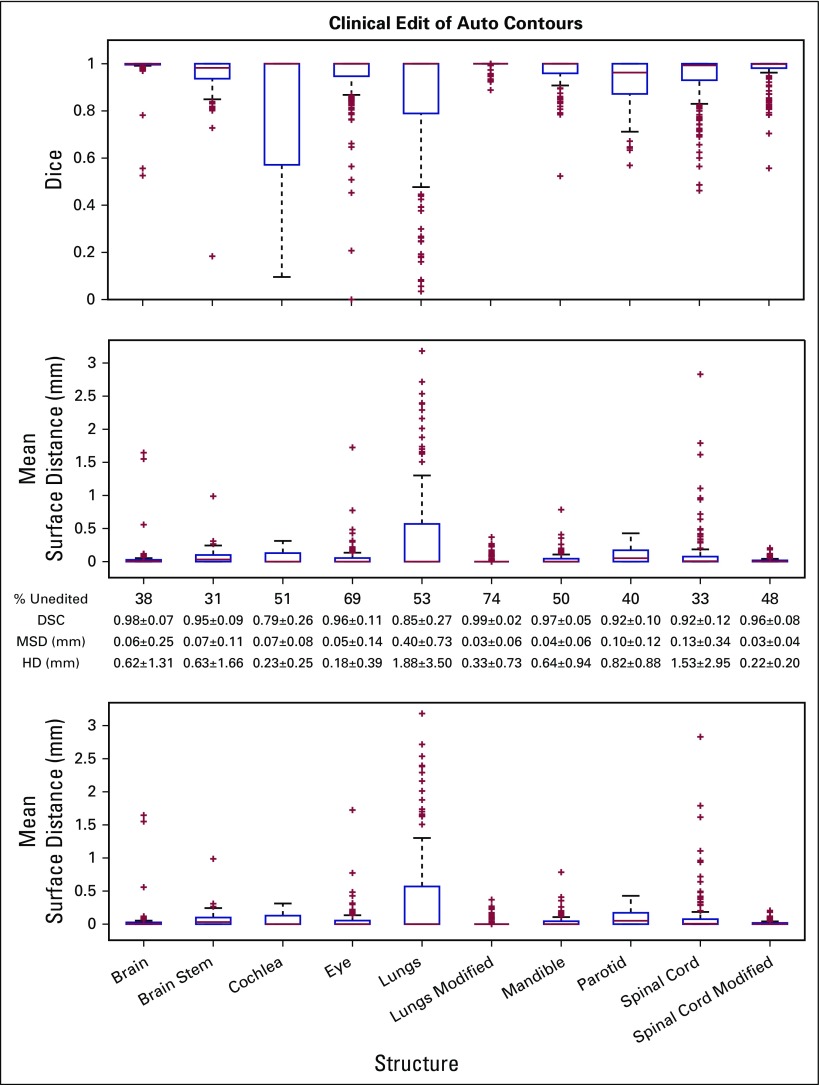
Distribution of the clinical edits to the autocontours. The red line within the box plot represents the median, and box edges represent the 25th and 75th percentile. Outliers are indicated by red crosses and are values outside the 25th or 75th percentile by more than 1.5 times the interquartile range. Between the box plots are the percent of unedited contours and the means (± standard deviation) of the Dice similarity coefficients and the mean surface distances for each for the automatically contoured or modified structures. DSC, Dice similarity coefficient; HD, Hausdorff distance; MSD, mean surface distance.

Since clinical implementation of the automated contouring software, 22 radiation oncologists have used it to generate normal structure contours for 166 patients. The seven attending physicians who used the tool the most accounted for 23%, 15%, 14%, 9%, 7%, 7%, and 5% of the total use. The mean ± SD time required for generation of the autocontours was 11.5 ± 3.1 minutes when run on a Windows 2012-based personal computer with an 8-core Xeon E5-2697 v3 2.6-GHz central processing unit and 16 GB of memory. Multithread computing was again implemented. No oversight is required and thus it can occur simultaneously with other required treatment planning tasks. Figure 2 shows the distribution of autocontour edits, as measured by the DSC, MSD, and HD. Only contours remaining in the treatment plan at the time of treatment with the same naming convention were considered.

Notably, radiation oncologists did not edit 49.8% of the contours for treatment planning. As shown in Figure 2, 31% of automatically contoured brainstems, 40% of parotids, and 48% of modified spinal cords were not edited for clinical use. The structures edited least often were the eyes (69%) and modified lungs (74%). The maximum MSD was seen for the unmodified lung (3.29 mm) and unmodified spinal cord (2.65 mm). As before, considering the modified lung avoidance and spinal cord structures, the MSDs were decreased nearly 10-fold to 0.33 mm and 0.20 mm, respectively.

## DISCUSSION

This study demonstrates the clinical feasibility of high-quality automated contouring of normal structures relevant for HNC radiation treatment planning and points toward its applicability in low-resource settings by saving time and removing a major hurdle when transitioning to more advanced RT techniques. This study represents, to the best of our knowledge, the first implementation of an automated contouring process for a large structure set in the head and neck. Retrospective quantitative comparison of autocontours with clinically used contours, ratings from multiple international radiation oncologists and, most importantly, the clinical implementation of automated contouring in a robust clinical practice shows the promise of autocontouring techniques.

In this study, we focused on organs at risk with safety implications (eg, spinal cord and brainstem) or broadly acknowledged toxicity benefit (eg, parotid glands). Other normal tissue structures can be relevant in certain settings, especially for toxicity avoidance (eg, early-stage cancers), including the submandibular glands, optic structures, esophagus, and larynx. These will be the subject of further improvement and be integrated into planning in future studies.

Before the clinical implementation of the software, the algorithm was validated internally and externally. Contours on a subset of patients were rated by both the internal primary physician and by multiple international physicians. Generally, we observed agreement between physicians, but consistent with previous findings,^10,11^ we also observed some interobserver variability. Ninety-three percent received scores from both physicians who grouped the contour into needing either no or minor edits, which indicated that each physician may have slightly different criteria for determining when minor edits are needed. Furthermore, the discrepancy between ratings may have arisen because the atlas contours were based on contours drawn or approved by the primary physician. Considering the large variability in physicians’ contouring practices, these results are not surprising.

Upon clinical implementation, 95% of contours had a maximum distance to edit (HD) of 2 mm, indicating that for the vast majority of patients, only minor edits were required for use in treatment planning. Discussions with the attending physicians revealed a perceived time benefit and, although the time needed to edit the autocontours was not an aim of this study, the study has established that the time required for editing an autocontour is less than that needed to generate the contour by hand.^28-30^ Since its clinical implementation, 22 attending physicians have used the automated contouring software, and nearly half the autocontours were not edited for use in the treatment plan. An investigation of the potential dosimetric impact of these clinical edits is ongoing.^31^ Used with or without edits, automated contouring solutions promise to be a pivotal part of the efforts to improve efficiency of RT planning in resource-constrained settings.

There are limitations for this analysis and more broadly for the potential utility of this approach. First, the contours used in the atlas were delineated by a single physician (although they were reviewed by a team of dedicated head-and-neck radiation oncologists), and the delineation of normal structures on the atlas patients will be propagated to test patients. Therefore, systematic differences in contouring practices between physicians may lead to edits. Although a single contouring atlas was used for this study, future work will determine whether additional center- or region-specific atlases may be required. Second, some physicians modify the normal structures away from base anatomy because of the need to cover adjacent areas with target dose. It is our opinion that true anatomy should be contoured accurately, with potential substructures created for planning (for instance, parotid planning target volume) and not to adjust the normal structure itself; however, some of our discrepancies are likely related to this. Data were not collected to determine the motivation for any clinical edit of autocontours.

Finally, the implementation of such a tool must be accompanied by proper training, cost assessment, and safety procedures.^32^ All autocontours must be thoroughly reviewed and approved by the physician before planning treatment. Quality assurance of the treatment and accessory equipment, of the treatment planning process, of the delivery, and of the quality assurance process itself, must not be overlooked. Many guiding documents are available that discuss accepted quality assurance processes,^33-35^ and any change of practice must be accompanied by a reassessment of these guiding principles. Automatic contouring requires the watchful eye of a trained clinician and vigilance to ensure automatic contouring is used safely and effectively. This article describes our initial work at developing, validating, and integrating the automated contouring of normal structures for HNC cases into clinical practice. This approach promises to improve efficiency in resource-constrained settings, in which transition to intensity-modulated RT planning may be prohibitive because of the time constraints of contouring multiple normal structures. More importantly, it serves as a crucial part of the larger attempt to automate RT planning for low- and middle-income countries to improve availability, efficiency, and quality of care worldwide. The next step in this work is the development of automated contouring of nodal targets, which would require the physician to delineate only the gross tumor volume and select which nodal levels are at risk. This has the potential to reduce the extensive variability in the delineation of target volumes, as noted in many previous investigations.^36^ Our results suggest that this approach is feasible, reproducible, and acceptable to an international group of physicians. Automation, including that of the contouring process, continues to be a promising avenue to relieve part of the staffing burden, time, and cost required for RT deployment in low-resource settings.
